# Dystrophin Protein Quantification as a Duchenne Muscular Dystrophy Diagnostic Biomarker in Dried Blood Spots Using Multiple Reaction Monitoring Tandem Mass Spectrometry: A Preliminary Study

**DOI:** 10.3390/molecules27123662

**Published:** 2022-06-07

**Authors:** Refat M. Nimer, Khalid M. Sumaily, Arwa Almuslat, Mai Abdel Jabar, Essa M. Sabi, Mohammad A. Al-Muhaizea, Anas M. Abdel Rahman

**Affiliations:** 1 Department of Medical Laboratory Sciences, Jordan University of Science and Technology, Irbid 22110, Jordan; 2 Clinical Biochemistry Unit, Pathology Department, College of Medicine, King Saud University, Riyadh 11461, Saudi Arabia; ksumaily@ksu.edu.sa (K.M.S.); esabi@ksu.edu.sa (E.M.S.); 3 Clinical Biochemistry Unit, Laboratory Medicine, King Saud University Medical City, King Saud University, Riyadh 11461, Saudi Arabia; 4 Metabolomics Section, Department of Clinical Genomics, Center for Genome Medicine, King Faisal Specialist Hospital and Research Center (KFSH-RC), Zahrawi Street, Al Maather, Riyadh 11211, Saudi Arabia; arwaalmeslat@hotmail.com (A.A.); n3502716@kfshrc.edu.sa (M.A.J.); 5 Department of Biochemistry and Molecular Medicine, College of Medicine, Alfaisal University, Riyadh 11533, Saudi Arabia; 6 Department of Neurosciences, King Faisal Specialist Hospital and Research Centre, Riyadh 11211, Saudi Arabia; mmuhaizea@kfshrc.edu.sa

**Keywords:** dystrophin, Duchenne muscular dystrophy (DMD), diagnostic biomarker, liquid chromatography–tandem spectrometry (LC–MS/MS), multiple reaction monitoring (MRM), dried blood spot (DBS)

## Abstract

Duchenne muscular dystrophy (DMD) is an X-linked recessive disorder characterized by progressive muscle loss, leading to difficulties in movement. Mutations in the DMD gene that code for the protein dystrophin are responsible for the development of DMD disorder, where the synthesis of this protein is completely halted. Therefore, circulating dystrophin protein could be a promising biomarker of DMD disease. Current methods for diagnosing DMD have sensitivity, specificity, and reproducibility limitations. Herein, a quantitative liquid chromatography–tandem spectrometry (LC–MS/MS) technique in multiple reaction monitoring (MRM) mode was designed and validated for accurate dystrophin protein measurement in a dried blood spot (DBS). The method was successfully validated on the basis of international guidelines regarding calibration curves, precision, and accuracy. In addition, patients and healthy controls were used to test the amount of dystrophin protein circulating in DBS samples as a potential biomarker for DMD disorders. DMD patients were found to have considerably lower levels than controls. To the best of our knowledge, this is the first study to report dystrophin levels in DBS through LC–MS/MS as a diagnostic marker for DMD to the proposed MRM method, providing a highly specific and sensitive approach to dystrophin quantification in a DBS that can be applied in DMD screening.

## 1. Introduction

Duchenne muscular dystrophy (DMD) is an X-chromosome-linked recessive disorder that affects one in every 3500–6000 live male births [1,2]. It is caused by gene mutations that code for the 427 kDa protein dystrophin [3]. As an organizing center for the dystrophin-associated protein complex (DAPC), dystrophin is critical for mechanical force transduction and is involved in signaling functions [4,5]. In addition, dystrophin connects the muscle fibers’ cytoskeleton to the extracellular matrix via its amino-terminal and carboxy-terminal domains, stabilizing the muscle fibers during movement [6]. Frameshifting mutations cause the premature truncation of protein translation in DMD, or nonsense mutations result in non-functional and unstable dystrophin [7]. Dystrophin deficiency is associated with skeletal and cardiac muscle weakness and atrophy due to progressive muscle degeneration and wasting [8]. At almost 5 years old, most patients are diagnosed, and they are wheelchair-bound by the time they reach adolescence. Unless an intervention is made, the average age of death is around 19 years old [9].

An early and accurate DMD diagnosis is essential for effective disease management. Currently, genetic tests are the widely used method for screening for DMD. Dystrophin gene deletion and duplication testing is usually the first confirmatory test because approximately 70% of patients with DMD have a single-exon or multi-exon deletion or duplication in the dystrophin gene. Multiplex ligation-dependent probe amplification (MLPA) [10] or comparative genomic hybridization array [11] are the best options for testing because multiplex PCR can only detect deletions. However, if deletion or duplication testing is negative, genetic sequencing should be performed to look for the remaining types of mutations associated with DMD (roughly 25–30%) [12]. Point mutations (nonsense or missense), small deletions, and small duplications or insertions are among the mutations that can be detected using next-generation sequencing [13]. Considering the gene’s size and the complexity of the genetic analysis, this rarely confirms the clinical diagnosis of DMD disorder in a timely fashion. In this case, a muscle biopsy sample should be tested for the presence of dystrophin protein using immunohistochemistry of tissue cryosections or a Western blot of a muscle protein extract [14].

Nonetheless, genetic tests have limitations due to genetic heterogeneity in DMD or technical issues, such as sensitivity and experimental design [15]. Moreover, Western blot and immunohistochemistry results have a low level of reproducibility because they can produce a large amount of inter-laboratory variation, especially with samples nearing the lower limit of quantification (LLOQ) [16,17].

Recently, the presence of circulating dystrophin protein in the blood of DMD patients has been the focus of only a few studies compared to the several studies focused on identifying protein biomarkers other than dystrophin [18,19,20]. Dystrophin C-terminal isoforms in serum such as Dp71 and Dp116 may be an indicator of DMD because it was found that patients with DMD had higher serum levels of some dystrophin isoforms when compared to healthy individuals [20]. Additionally, a single case of an exons 5–44 in-frame deletion suggested that serum from patients with in-frame deletions could contain dystrophin molecules that had been deleted internally [20]. However, methods based on measuring dystrophin protein concentrations in biological samples face technical constraints, such as sensitivity, because dystrophin protein concentrations are very low in healthy and diseased muscles.

Furthermore, DMD is often undiscovered until the late pre-school years, so DMD diagnosis is delayed by 2–5 years on average [21]. Therefore, there is an urgent need for alternative diagnostic methods for screening and diagnosing DMD.

Mass-spectrometry-based targeted proteomic methods, such as multiple reaction monitoring MRM, are emerging as viable tools for confirming candidate proteins in biological and biomedical applications [22]. MRM is a highly specific and sensitive mass spectrometry technique that uses triple quadrupole (QQQ) MS equipment to quantify selected analytes such as proteins in very complex samples [23,24].

As a molecular substitute for the respective intact proteins, MRM measures the concentrations of peptides produced by the enzymatic digestion of targeted protein [25,26]. This method uses two mass analyzers, one to select peptide ions that fall within an acceptable mass range and one to fragment the parent peptide ions using collision-activated dissociation (Q2). One or more fragment ions generated by dissociation can then be measured by the second mass analyzer (Q3) [27]. The absolute quantitation of proteins is achieved by incorporating stable isotopes into quantitative proteomic workflows then comparing the mass spectrometric signal of peptides present in the sample with signals from synthetic peptides [28,29,30]. Although it is necessary to conduct extensive method development in order to identify the most specific and sensitive transitions for each targeted peptide targeted by MRM-based quantification methods, MRM assays have a high sensitivity and specificity for protein quantification over a wide dynamic range of concentrations, minimizing non-biological variations [31].

This study aimed to quantify the dystrophin protein in a DBS by utilizing liquid chromatography–tandem mass spectrometry in MRM mode (LC–MS/MS) with selected dystrophin signature peptides as measurement surrogates. Furthermore, we aimed to establish clinical reference ranges for dystrophin on the basis of the concentrations of dystrophin-selected signature peptides in a DBS.

## 2. Results and Discussion

### 2.1. Signature Peptide Selection and LC–MS/MS Method Development

The human UniProt-FASTA database was used for obtaining protein sequences and isoforms of dystrophin protein (UniProt ID: P11532). The selection of signature peptides was obtained in subsequent in silico tryptic digestion of dystrophin protein using PeptideMass and validated using Skyline Software (version 19.1, Washington, DC, USA). The signature peptides of the dystrophin protein were selected on the basis of criteria outlined in the Section 3.

Table 1 summarizes the properties of selected signature peptides unique for the dystrophin protein. Dystrophin proteins were quantified using light and heavy (stable-isotope-labeled, SIL) signature peptides, used as the standard and IS, respectively, for the absolute quantification process. A calibration curve for measuring the peptide signal was created using standard reference material. The isotopically labeled peptides, also known as heavy peptides, have the same amino acid sequence and physicochemical characteristics as light peptides. This means that the ionization efficiency of peptides must be the same in the unknown sample and the calibration curve [32]. This work compensated several analytical differences, such as ionization efficiency and ion suppression, using isotopically labeled copies of each peptide as ISs that co-eluted at the same retention period. The peptide concentrations were measured by comparing the light (analyte) signals to the heavy (reference) signals (IS) [33].

The LC–MS/MS method was optimized by using a mixture of the four signature peptides and their labeled analogs. First, the MRM transitions, such as the ion source and the triple quadrupole analyzer, were optimized to produce the most abundant precursor and product ions for every peptide at the optimal cone voltage and collision energy. The cone voltage ranged from 26 to 46 V, and the collision energy was between 48 and 80 eV. Table 1 summarizes the optimal LC–MS/MS method parameters such as retention time (RT), precursor ion (Q1), and product ion (Q2).

### 2.2. Validation of LC–MS/MS Method

Bioanalytical methods validation was validated according to the U.S. Food and Drug Administration (FDA) guidelines [34].

#### 2.2.1. Curve Linearity

Calibration curves for each of the two selected dystrophin signal peptides were used to define the protein measurement range and linearity.

**Figure 1:**
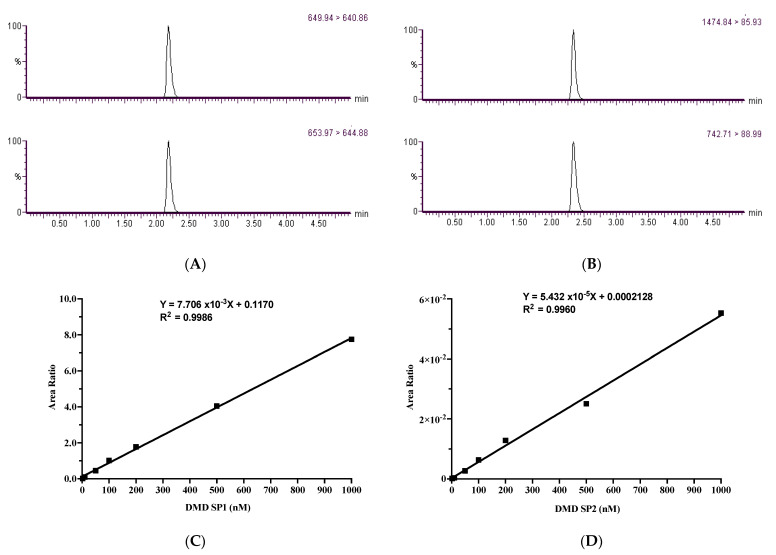
Representative extracted ion chromatography (EIC) and signature-peptide-based calibration curves. (**A**) Extracted ion chromatograms for the dystrophin SP1 (649.94 > 640.86) (quantitative transition) and its labeled internal standard transition (653.97 > 644.88). (**B**) EIC of the quantitative transition for dystrophin SP2 (1474.84 > 85.93) and its internal standard (742.71 > 88.99). (**C**) Calibration curve of the dystrophin SP1 and (**D**) dystrophin SP2.

Figure 1A,B represents the extracted ion chromatograms of the dystrophin SP1 and SP2, as well as their labeled internal standards. In both dystrophin SP1 (Figure 1C) and dystrophin SP2 (Figure 1D), the calibration curves were linear over the range of 2.0 to 1000 nM, having good linearity with correlation coefficients (R2) of 0.9986 and 0.9960, respectively.

#### 2.2.2. Specificity and Sensitivity

The method’s sensitivity was assessed as described in the Section 3, with the results summarized in Table 2. LLOQ was tested in triplicate on three different days for both signature peptides. The LLOQ was found to be 2.0 nM. Furthermore, the linearity of the method was assessed for each of the two signature peptides, with coefficients of determination (R2) of 0.9991 and 0.9931, respectively. The absence of interferences with the same MRM transition and retention times was defined as specificity. No carryover residues were observed from the carryover experiment described in the Section 3.

#### 2.2.3. Inter- and Intraday Validation

In order to assess the precision and accuracy, six independent runs of LQC, MQC, and HQC samples were carried out on the same day (intraday) and three other days (interday).

There was less than 15% interday variability and 80–120% interday accuracy for the chosen signature peptides (Table 3) for QCs, which was in line with the acceptable limits of the ICH [35,36]. Intra- and inter-day validation demonstrated good accuracy and precision.

#### 2.2.4. Peptide Stability Study

QC samples at three concentrations were used to evaluate the stability of the two DMD signature peptides in three replicated experiments (25, 150, and 750 nM). Stability tests were performed under a range of different storage settings, including RT for 24 h, 4 °C for one week, –20 °C for two weeks, and –20 °C for one month. The two chosen signature peptides’ stabilities varied from 86.5% to 141.2% (Table 4).

### 2.3. Dystrophin Protein Absolute Quantification in Clinical Samples

The LC–MS/MS method has limitations in selectivity due to “isobaric” interferences, known as the “ion suppression effect”. However, despite these limitations, the LC–MS/MS method is the most robust analytical technique for quantifying proteins that provide high sensitivity and accuracy analysis [33].

#### 2.3.1. Diagnostic Evaluation

**Figure 2:**
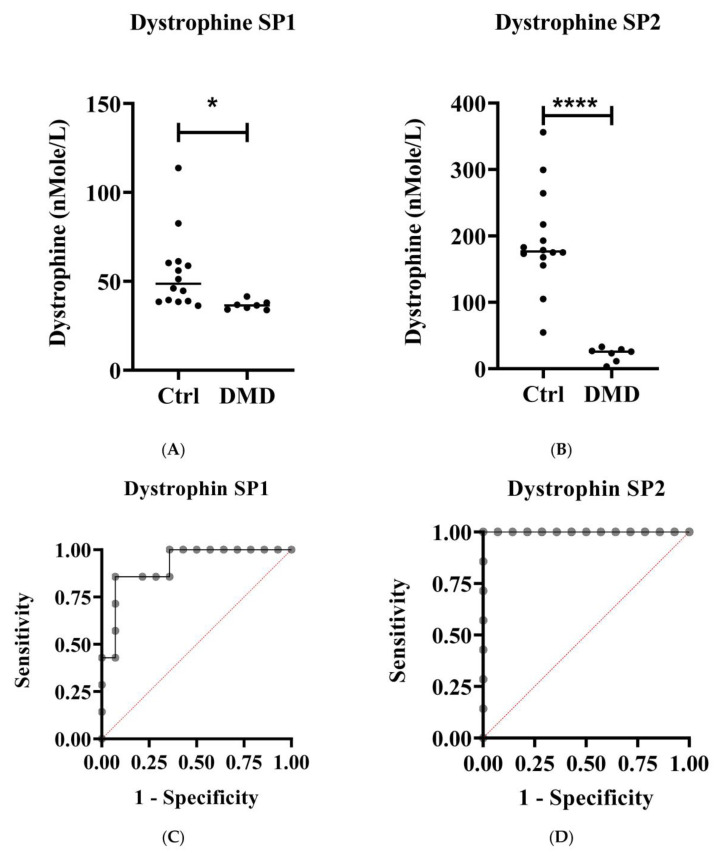
Dystrophin protein level in dried blood spot (DBS). The dystrophin protein level in DMD patients and healthy controls (Ctrl) was based on (**A**) dystrophin SP1 and (**B**) dystrophin SP2. The sensitivity and selectivity of CF based on these signature peptides were evaluated using (**C**) ROC curve for dystrophin SP1 with AUC 0.9184 (99% CI, *p*-value 0.0022) and (**D**) dystrophin SP2 with AUC 1.0 (99% CI, *p*-value 0.0001). The dashed red lines represent the random classifiers (x = y), above which an instance is classified as positive, and below which, it is negative. * *p* < 0.05, **** *p* < 0.0001.

**Figure 3:**
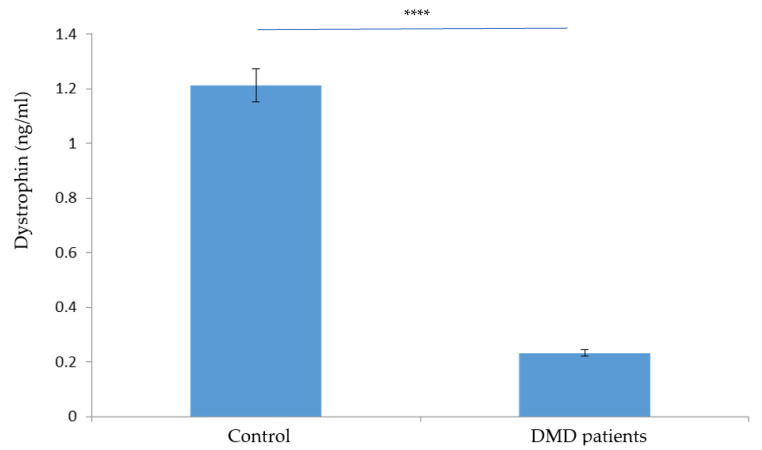
Dystrophin protein level in a DBS. The dystrophin protein level in DMD patients and healthy controls (Ctrl) by ELISA. The difference is statistically significant (****).

Dystrophin SP1 and SP2 were used to determine the absolute protein concentration. DMD protein levels in the patients’ group were significantly lower than those of the control group (*p* < 0.05) (Figure 2A,B). MRM assay results were confirmed using ELISA (Figure 3).

The diagnostic value of the DMD protein based on SP1 and SP2 was assessed using receiver operating characteristic (ROC) curves (Figure 2C,D). The true positive rate (sensitivity) was plotted on the y-axis, while the false positive rate (specificity) was plotted on the ROC curve. The dashed red lines represent the random classifiers (x = y), above which an instance is classified as positive, and below which it is negative. As a result, the “ideal” point lies at the extreme top-left-hand corner of the ROC plot, with zero false positives and one true positive. A perfect test regarding sensitivity and specificity will have a value of area under the ROC curve (AUC) greater than 1.0. In both the control and DMD patients’ groups, the AUCs (Figure 2C,D) for the dystrophin SP1 and SP2 were 0.9184 (99% CI, *p*-value 0.0022) and 1.0 (99% CI, *p*-value 0.0001), respectively. As a result, dystrophin SP2 demonstrated higher diagnostic and analytical performance in distinguishing between DMD patients and healthy controls than SP1.

#### 2.3.2. Reference Range Determination

Table 5 shows the mean value, standard deviation, standard error of the mean, minimum/maximum values, and 95% reference intervals for the dystrophin protein based on the dystrophin signature peptide concentrations in the DBS.

The clinical reference range for dystrophin protein based on selected signature peptide concentrations in a DBS was determined by following the recommendations of the Clinical and Laboratory Standards Institute (CLSI) and the International Federation of Clinical Chemistry (IFCC) [37,38]. Accordingly, dystrophin protein levels were measured on the basis of SP1 and SP2 in control and DMD patients (*n* = 17 and *n* = 10, respectively) (Table 5).

Dystrophin levels based on SP1 ranged between 42.60 and 62.93 nM for control and 14.82 to 38.15 nM for DMD patients (Table 5), whereas the dystrophin level based on SP2 was between 120.2 and 212.5 nM for control and between 7.234 and 25.12 nM for DMD patients (Table 5). As a result, dystrophin values were considered normal according to SP1 and SP2 of >42.60 nM and >120.2 nM, respectively. 

The variation in dystrophin levels between SP1 and SP2 in patients with DMD and control groups is due to the site of SP1 and SP2 and the accessibility for an efficient digestion by trypsin.

The quantification of dystrophin by MRM is not based on the whole sequence of protein but a small sequence (signature peptide) after clearing the sequence of a signature peptide from any potential genetic mutation. Therefore, patients might have a trace level of these signature peptides generated from the trypsin digestion of the dystrophin.

These findings are considered preliminary; hence, at least 120 data points are needed for a reference interval study [39].

## 3. Materials and Methods

### 3.1. Materials Used

All dystrophin signature peptides, SP1 and SP2 unlabeled “light” and labeled “heavy” (D8 labeled Val and D3 labeled Leu), were custom synthesized by Genemed Synthesis, Inc. (San Antonio, TX, USA). RapiGest SF surfactant was purchased from Waters Corporation #186001860 (Milford, MA, USA). Dithiothreitol (DTT), iodoacetamide (IAA), formic acid (FA), ammonium bicarbonate (ABC), methanol, and acetonitrile (ACN) were purchased from Sigma-Aldrich (St. Louis, MO, USA).

### 3.2. Subjects and Blood Samples

Whole blood from genetically confirmed DMD patients (*n* = 7) and gender- and age-matched healthy control (*n* = 17) samples were collected in EDTA tubes. Blood was then spotted on newborn screening filter papers (#226, PerkinElmer, Turku, Finland), which were dried immediately to obtain the DBS and then stored at −20 °C. 

King Faisal Specialist Hospital and Research Centre’s (KFSHRC) neurology department evaluated all patients clinically and genetically on the basis of an IRB approval (RAC#2170 001). The “patients’ group” consisted of 6 males and 1 female, confirming DMD deletion/duplication or point mutations (Table 6). Patients were excluded from this study due to (1) inability or unwillingness to provide informed consent, or (2) diagnosis with conditions other than DMD. The healthy control samples were collected from our biobank by matching the study age and gender, where their clinical details were kept blinded as collected per the IRB approval of establishing this biobank.

### 3.3. Signature Peptide Selection

The National Center for Biotechnology Information (NCBI) protein database was used to identify the protein sequence of human (Homo sapiens) dystrophin, with the accession number P11532. The in silico digestion with trypsin of dystrophin was performed by the PeptideMass tool, an Expasy bioinformatics resource portal of the Swiss Institute of Bioinformatics, in order to obtain a list of possible signature peptides [35]. The resulting sequences were subjected to Basic Local Alignment Search Tool (BLAST) searches of the NCBI protein database against other protein sequences to verify their uniqueness to the target protein [35].

Moreover, the selection of signature peptides was based on (1) the length of the peptide (5–25 amino acids); (2) peptide water solubility using Innovagen’s peptide calculator (PepCalc.com); (3) the stability of peptides using the ProtParam tool (http://web.expasy.org/protparam/, accessed on 17 May 2022); (4) alignment to all dystrophin isomers performed using the SIM Alignment Tool for protein sequences, a bioinformatics resource portal maintained by the Swiss Institute for Bioinformatics (SIB); (5) post-translational modifications (PTMs) including methylation, glycosylation, and phosphorylation, with a risk of methylation, checked manually and for other PTMs by using the UniProt and Phospho. ELM databases [33,36,37,40]. As a result, SP1 (QVASSTGFCDQR) and SP2 (LGLLLHDSIQIPR) were identified as two signature peptides for dystrophin protein. For technique development, these two peptides were produced. To be utilized as standard and internal standards, the selected signature peptides were synthesized as light and heavy (stable-isotope-labeled (SIL)) signature peptides (IS).

The study standard materials were provided with purity > 95% by high-performance liquid chromatography (HPLC). The peptide sequence identification, mainly the labeling location, was depicted using a high-resolution mass spectrometry (HRMS) analysis. The quality of the standard materials is certified by the supplier, where an HPLC chromatogram and MS spectra are attached to each standard material.

### 3.4. MRM Transition Development for Analytes

#### 3.4.1. Stock and Working Solution Preparation

In deionized water (dH_2_O), each peptide’s 2.0 mg/mL stock solution, unlabeled and labeled, was prepared. For mass spectrometric tuning and chromatographic optimization, 20.0 µg/mL working solutions in the mobile phase (90:10 ratio of 0.1% FA in H_2_O/0.1% FA in ACN) were produced from the stock solution.

#### 3.4.2. LC–MS/MS Analysis

The LC–MS system (Waters Corporation’s XEVO TQ-MS) was used in the electrospray positive-ionization (+ESI) mode for signal optimization, wherein 20.0 ug/mL of each peptide (labeled and unlabeled) working solution was infused into the mobile phase (90:10 H_2_O/ACN, 0.1% FA) with a flow rate of 20 µL/min. The desolvation temperature was 350 °C, whereas the ion source temperature was 150 °C. The capillary and cone voltages were set at 3.90 kV and 23 V, respectively, to detect the peptide’s precursor ion. During the tuning process, the spraying gas flow rate was kept constant at 500 L/h. After obtaining each peptide’s precursor ion data, an automated tuning was optimized for each transition using Xevo-IntelliStart (Waters, Milford, CT, USA) to determine the product ions, cone voltage, and collision energy. In the MS analyzer, the optimal parameters for each analyte were utilized for MRM transitions, where at least two transitions were established for each analyte. Following the construction of the MRM transitions, the targeted peptides were separated using ultra-high-pressure liquid chromatography (UPLC) ACQUITY UPLC BEH C18, 1.7 m, 2.1 mm 50 mm column at 25 °C using a gradient mobile phase as described previously [41]. The peptide mixture solution was first injected at a flow rate of 0.300 mL/min over a total of 10 min of run time. The gradient profile for mobile phase A (0.1% FA in H_2_O) was 90%. After 2 min, mobile phase A reaches 10% until the original ratio returns to 90% and 10% for A and B (0.1% FA in ACN), respectively, at 5 min. Finally, the column was equilibrated over 10 min at 10% solvent B before we performed a second injection. An Acquity Ultra-High-Pressure Liquid Chromatography UPLCXEVO TQD Triple-Quadrupole-Tandem Mass spectrometer (Waters Corporation, Milford, CT, USA) evaluated the calibrators as well as the quality control (QC) set of samples. The following general MS parameters were modified for LC–MS/MS: the desolvation temperature: 500 °C; the desolvation gas flow: 1000 L/h; the cone gas flow: 50 L/h; MS capillary source voltage: 1.98 KV; and the cone source voltage: 47 V. Following the gradient, each sample was run for 10 min at a mobile phase flow rate of 0.3 mL/min. For the injection, a volume of 10 mL was injected after samples were kept in the autosampler at 4 °C. Sample carryover was minimized by applying frequent washing cycles.

### 3.5. DBS Sample Preparation

#### 3.5.1. Protein Elution and Trypsin Digestion from DBS

Five disks (3.2 mm each) were cut out and suspended in 200 μL (0.1% RapiGest SF) surfactant from each subject’s DBS sample and dissolved in 50 mM ABC at pH 7.8. Once the proteins were dissolved, they were incubated in a Thermomixer. Disulfide bonds of proteins were reduced by using 10 mM DTT. After that, samples were incubated at 60 °C for 30 min. Proteins were alkylated by 10 mM IAA at room temperature in the dark for 40 min. After that, this solution was subjected to trypsin digestion (prepared in 50 mM of ABC, pH 7.8) at a final ratio of 1:20 enzyme/protein, and incubated overnight at 37 °C. The remaining RapiGest materials were removed by adding 100% FA (pH ≈ 2) to the solution. Then, the samples were vortexed and incubated for 30 min at 80 °C. Finally, solid-phase extraction was performed on the digested peptides (SPE) following centrifugation of the samples and collecting the supernatant at 14,000 rpm for 20 min.

#### 3.5.2. Solid-Phase Extraction

The tryptic peptides were purified with solid-phase extraction (SPE). The cartridges (Waters Oasis PRiME, Waters, Millford, MA, USA) were set under vacuum and conditioned with 100% methanol, followed by dH_2_O. The tryptic digested samples were mixed with labeled peptides (IS) and loading buffer (0.1% FA in dH_2_O) to dilute the samples before passing through the sorbent. The unwanted impurities were washed out twice with dH_2_O. The targeted peptides were eluted with 1.0 mL 0.1% FA in 50% ACN and dried using a Savant^TM^ DNA Concentrator SpeedVac (Thermo Scientific, Asheville, NC, USA). For LC–MS/MS analysis, the dry extracts were reconstituted in 100 μL mobile phase (90:10) 0.1% FA in H_2_O/0.1% FA in ACN.

### 3.6. LC–MS/MS Method Validation

MRM transitions were developed for the dystrophin peptides. The method was validated following the Food and Drug Administration’s (FDA) and the International Conference on Harmonization’s (ICH) analytical method validation guidelines [34,35].

#### 3.6.1. Curve Linearity

The calibration curves and QC samples in a biological matrix were generated using a 20 µg/mL mixture of unlabeled peptides. For the linearity test, three calibration curves were generated each day for three days in a row. Calibration curves were generated for the two peptides and plotted in y-axis proportion to the nominal concentration of the standard as area ratios (area of standard/area of internal standard, IS) (x-axis). Calibration curves were tested for linearity by calculating the correlation coefficient (R2), which should be less than 0.98.

#### 3.6.2. Specificity and Sensitivity

The three-day calibration curves determined the limits of detection (LOD) and lower limits of quantification (LLOQ) for each analyte. LLOQ was established as the lowest point on the calibration curve, and it has to be at least 10 times the standard deviation of the blank. S/N of at least 3 and 10 is required for the LOD and LLOQ concentrations, respectively. The LLOQ should have an accuracy range of 80–120% and a daily variance of <20%. Thus, the LLOD was adjusted with an S/N ratio of ≥3.

#### 3.6.3. Inter- and Intraday Validation

The three QC concentrations’ repeatability (intraday precision) and reproducibility (interday precision) were examined to determine the approach’s precision and accuracy. On the same day (intraday), six replicates of low-quality control samples (LQC), medium-quality control samples (MQC), and high-quality control samples (HQC) were analyzed for reproducibility determination and on three different days (interday) for reproducibility assessment. A percentage of relative standard deviation was used to assess signal variability (percent RSD). The interday precision for each QC was determined using the following formula: (σ of measured QC concentrations on day x/mean of measured concentrations on day x) 100%. Additionally, the method estimated the intraday precision (σ of measured QC concentrations over three days/mean of measured QC concentrations over three days) 100%. As a result, the difference between one day and the next should be 20%. Interday accuracy of each QC was calculated as (mean measured concentration /nominal concentration) × 100%. Intraday accuracy was calculated on the basis of the following formula: (mean measured concentration over three days/nominal QC concentration) × 100%. For all other standard levels in the calibration curve, the accuracy was adjusted at an acceptable range of 80–120%.

#### 3.6.4. Peptide Stability Study

The storage stability for two selected signature peptides of dystrophin was evaluated by assessing sets of three QC samples for four weeks under different preparation and analysis circumstances, including room RT, benchtop, in an autosampler, in a refrigerator (4 °C), and in a freezer (–20 °C). Stored QC samples were tested against freshly prepared QCs. For each QC level, the molecular stability was determined by (average area ratio of examined sample/average area ratio of the fresh sample) × 100%.

#### 3.6.5. Carryover

The assay carryover was evaluated by injecting the highest standard 6 times, followed by 3 blank samples.

### 3.7. Quantification of Dystrophin Protein by Sandwich Enzyme-Linked Immunosorbent Assay (ELISA)

The proteins extracted from 5 punches (3.2 mm each) of DBS samples were analyzed by following the manufacturer’s protocols of the ELISA kits. Briefly (MBS2500550, MyBioSource (San Diago, CA, USA)), 100 µL of the DBS extracts and the standard solutions were incubated in the antibody pre-coated wells for 2 h at 37 °C. After washing, 100 µL of biotin-conjugated secondary antibody was incubated for 1h at 37 °C. Then, the wells were washed and incubated with 100 µL of horseradish-peroxidase (HRP)-conjugated avidin for 30 min followed by rinsing the wells and incubating with the 3,3′,5,5′-tetramethylbenzidine (TMB) substrate solution for 15 min. Finally, the reaction was stopped by adding 50 µL Stop solution, and the ELISA plates were measured using a plate reader at 450 nm wavelength. Each sample was tested in duplicate.

### 3.8. Data and Statistical Analysis

The LC–MS/MS MassLynx software, version 4.1 (Waters Corporation, Milford, MA, USA), was used to acquire, process, and visualize the data.

Data for the quantitative signal are represented as mean ± standard deviation (SD). Student’s *t*-test was used to analyze the statistical significance of the differences between means of dystrophin protein measuring by ELISA and LC–MS/MS between DMD and healthy control subjects (GraphPad Prism software version 6.01). According to the figure legends, *p*-values of <0.05 were considered significant. GraphPad Prism software version 6.01 was used to prepare the graphs (Version 6.01; Graph Pad Software, La Jolla, CA, USA).

## 4. Conclusions

To the best of our knowledge, this is the first time the dystrophin protein has been precisely measured using an MRM mass spectrometry assay. To quantify dystrophin protein in DBS using selected signature peptides, we developed and validated an LC–MS/MS method that was both sensitive and accurate. We found that the dystrophin protein levels were reduced in DMD patients and could be used as a diagnostic marker for DMD in newborns. In the future, this method will be useful for determining the amount of dystrophin protein present in a DBS that can be applied in routine testing for DMD. This method is limited to a small number of patients with almost the same genetic variation. In order for the clinical feasibility of the method to replace the most invasive functional test, muscle biopsy, a larger group of patients should be recruited. A correlation between the DMD levels and the gene variation is crucial and a significant added value to this approach. Moreover, a study of the ability of this method to discriminate between DMD and Becker muscular dystrophy (BMD) patients in the context of dystrophin concentrations in the blood is needed.

## Figures and Tables

**Table 1:** List of dystrophin signature peptides and their internal standard details, including the instrumental conditions. The underlined valine and leucine moieties were labeled with d8 and d3, respectively.

DMD Signature Peptides	Quantification Property	Peptide Amino Acid Sequence	Molecular Weight (g/mol)	Purity (%)	Precursor Ion (*m*/*z*)	Product Ion (*m*/*z*)	Cone Voltage (V)	Collision Energy (v)	Retention Time (min)
Dystrophin SP1	Quant	QVASSTGFCDQR	1298.2	98.05	649.94	640.86	30	20	2.25
	Qual					182.5	30	44	
Dystrophin SP1-labelled	Quant	QVASSTGFCDQR (V: d8-Val)	1306.4	99.3	653.97	644.88	28	20	2.25
	Qual					190.36	28	40	
Dystrophin SP2	Quant	LGLLLHDSIQIPR	1475.0	98.15	1474.84	85.93	100	80	2.42
	Qual					109.95	100	78	
Dystrophin SP2-labelled	Quant	LGLLLHDSIQIPR (L: d3-Leu)	1483.5	98.99	742.71	88.99	42	54	2.41
	Qual					109.95	42	74	

**Table 2:** Linearity and sensitivity summary over three days.

Dystrophin Peptide	Linearity (*n* = 3)			LLOQ (nM)
	Slope	Intercept	R2	
Dystrophin SP1	0.0202	0.19353	0.9991	2.0
Dystrophin SP2	4.373 × 10^−5^	0.00083	0.9931	2.0

**Table 3:** Linearity and sensitivity summary over three days.

QC Concentration (nM)	Summary	Inter-Day Validation (*n* = 18)		Intra-Day Validation (*n* = 6)	
		Dystrophin SP1	Dystrophin SP2	Dystrophin SP1	Dystrophin SP2
25.0	Mean	26.41	25.78	25.86	25.49
	SD	3.69	5.20	4.74	3.30
	Accuracy %	105.62	103.11	103.43	101.95
	Imprecision CV%	14.42	19.60	18.33	12.93
150.0	Mean	159.79	143.96	155.76	146.32
	SD	35.41	31.86	4.68	5.87
	Accuracy %	106.53	95.98	103.84	97.55
	Imprecision CV%	22.02	22.19	3.01	4.02
750.0	Mean	833.47	751.79	732.71	753.25
	SD	157.41	27.82	109.71	32.33
	Accuracy %	111.13	100.24	97.70	100.43
	Imprecision CV%	18.42	3.70	14.97	4.29

**Table 4:** Dystrophin signature peptide solution stability at different storage conditions for a determined period.

Dystrophin SP	QC (nM)	Stability (%)				
		Fresh	RT	4 °C	Freezer (−20 °C)	
			24 h	1 Week	2 Weeks	1 Month
Dystrophin SP1	25	100.00	141.2	141.2	94.7	102.9
	150	100.00	104.9	104.9	95.6	98.8
	750	100.00	86.5	86.5	106.2	102.8
Dystrophin SP2	25	100.00	95.2	102.4	108.7	106.4
	150	100.00	119.4	108.5	107.3	105.9
	750	100.00	102.0	96.7	98.0	103.9

**Table 5:** Dystrophin protein clinical reference range in nM based on the dystrophin signature peptide concentration in a DBS.

Statistical Parameters	Dystrophin SP1		Dystrophin SP2	
	Ctrl	DMD	Ctrl	DMD
Number of values (*n*)	17	7	17	7
Minimum (nM)	36.24	2.280	37.90	2.280
Maximum (nM)	113.7	41.42	356.0	32.93
Range	77.46	39.14	318.1	30.65
95% CI of median				
Actual confidence level	95.10%	97.85%	95.10%	97.85%
Lower confidence limit	38.87	3.140	105.1	3.140
Upper confidence limit	58.79	37.94	192.9	29.14
Mean	52.77	26.49	166.3	16.18
Std. deviation	19.76	16.31	89.69	12.50
Std. error of mean	4.793	5.158	21.75	3.954
Lower 95% CI of mean	42.60	14.82	120.2	7.234
Upper 95% CI of mean	62.93	38.15	212.5	25.12

**Table 6:** DMD patients’ demographic details.

ID #	Gender	Age	DMD Mutation ^1^	Muscle Biopsy
DMD 1	M	12	exon 10 and 11 deletion c.650−?_1331?del	Yes
DMD 2	F	10	c.10141C>T; p.Arg3381 *	No
DMD 3	M	8	deletion of exon 49-50 (out of frame) c.7099−?_7309?del	No
DMD 4	M	10	duplication of exon 18 to 44 c.650−?_6438+?dup	No
DMD 5	M	10	c.2650C>T; p Gln884 *	Yes
DMD 6	M	5	exon 48 through 53 c.6913−? _7872? del	Yes
DMD 7	M	8	exon extension c.2862G>A; p.Trp954 *	No

^1^ The mutations are described according to the nomenclature derived by the Human Genome Variation Society (http:/www.hgvs.org, accessed on 17 May 2022). * stop codon.

## Data Availability

Not applicable.

## Sample Availability

Samples of the compounds are not available from authors.
